# Cell Visco-Elasticity Measured with AFM and Optical Trapping at Sub-Micrometer Deformations

**DOI:** 10.1371/journal.pone.0045297

**Published:** 2012-09-19

**Authors:** Schanila Nawaz, Paula Sánchez, Kai Bodensiek, Sai Li, Mikael Simons, Iwan A. T. Schaap

**Affiliations:** 1 III. Physikalisches Institut, Faculty of Physics, Georg-August Universität Göttingen, Göttingen, Germany; 2 DFG Research Center for Molecular Physiology of the Brain (CMPB)/Excellence Cluster 171, Göttingen, Germany; 3 Max-Planck Institute for Experimental Medicine, Göttingen, Germany; 4 Department of Neurology, Georg-August Universität Göttingen, Göttingen, Germany; University of Zurich, Switzerland

## Abstract

The measurement of the elastic properties of cells is widely used as an indicator for cellular changes during differentiation, upon drug treatment, or resulting from the interaction with the supporting matrix. Elasticity is routinely quantified by indenting the cell with a probe of an AFM while applying nano-Newton forces. Because the resulting deformations are in the micrometer range, the measurements will be affected by the finite thickness of the cell, viscous effects and even cell damage induced by the experiment itself. Here, we have analyzed the response of single 3T3 fibroblasts that were indented with a micrometer-sized bead attached to an AFM cantilever at forces from 30–600 pN, resulting in indentations ranging from 0.2 to 1.2 micrometer. To investigate the cellular response at lower forces up to 10 pN, we developed an optical trap to indent the cell in vertical direction, normal to the plane of the coverslip. Deformations of up to two hundred nanometers achieved at forces of up to 30 pN showed a reversible, thus truly elastic response that was independent on the rate of deformation. We found that at such small deformations, the elastic modulus of 100 Pa is largely determined by the presence of the actin cortex. At higher indentations, viscous effects led to an increase of the apparent elastic modulus. This viscous contribution that followed a weak power law, increased at larger cell indentations. Both AFM and optical trapping indentation experiments give consistent results for the cell elasticity. Optical trapping has the benefit of a lower force noise, which allows a more accurate determination of the absolute indentation. The combination of both techniques allows the investigation of single cells at small and large indentations and enables the separation of their viscous and elastic components.

## Introduction

Understanding the mechanics of cells has become increasingly important, since many cellular processes have been found to be regulated by, or linked to changes in the mechanical properties of the cell. Determining parameters such as the stiffness and the viscosity of cells is useful to understand cellular processes that involve mechanical changes and have been related to different conditions of the cell. Previously it was shown that during differentiation of cells, but also during the cell cycle, morphological changes of cells are in part governed by cell mechanics [1], [2]. Also, distinct mechanical properties have been measured for different cell types, which can be related to their specific roles in a tissue [3]. This relation can be employed to distinguish for example cancer cells from their healthy counterparts [4], [5]. Furthermore, cells respond to the composition and stiffness of the surface which they are cultured on, and show a reduced stiffness when grown on soft substrates [6], [7]. These findings indicate that both mechanical and biochemical signals act in a concerted way to define the cellular response upon stimuli.

Animal cells have a highly complicated architecture with a plasma membrane that is relatively inextensible and supported by a ≈100 nm thin cortical layer. This cortical network is composed of actin filaments, actin-binding proteins including myosin motors, and encloses a crowded liquid environment, the cytoplasm. The different components of the cell all contribute to the cell-mechanical response, but in a manner that depends on the measurement technique and timescale of the experiment. A variety of techniques have successfully been applied to measure the mechanics of single cells, including atomic force microscopy (AFM) [8], [9], magnetic twisting cytometry (MTC) [10], [11], micropipette aspiration [12], microplate cell manipulation [13], [14], optical stretchers [15], [16], particle tracking microrheology [17], [18] and optical traps [19], [20].

AFM which employs a probe to indent the cell, is often the method of choice to quantitatively measure the cell’s stiffness. As compared to other techniques the contact between the AFM probe and the cell can be reasonably well defined when the cell is indented in an almost vertical direction, normal to the coverslip. By using the Hertzian contact model, this symmetrical geometry of the experiment allows the extraction of the elastic Young’s modulus [9]. Conventional AFM tips are very sharp (<30 nm radius), which results in a high local stress on the cell. When the indentation is performed at nano-Newton forces this is likely to induce damage, which may have an effect on the measured results. The lowest force that can be exerted is limited by the thermal noise of the AFM cantilever in liquid, which is around 20 pN [21]. In practice most AFM experiments are performed from 0.1 nN up to a few nN. This force-noise also limits the accuracy at which the absolute cell indentation can be measured.

All of the aforementioned techniques have been applied to measure the mechanical response of cells at different loading rates (rheology). Although the variation in reported values for the absolute cell stiffness is large, most studies agree that cells respond stiffer when probed at higher frequencies. More recently it was recognized that the majority of the rheology experiments show that the cell stiffness (*k*) does not depend linear on the frequency (*f*) but obeys a weak power law: *k(f)∼ f ^α^* [8], [10], [11], [22]. The value of the exponent *α* lies in the range 0.1–0.35, and depends on the part of the cell that is probed [16], [17], [23], which make *α* a useful parameter to mechanically identify different structures of the cell.

Forces below the 20 pN force limit of AFM can be applied with MTC, optical stretchers and optical traps. The optical trap, where a focused laser beam is used to hold a micrometer sized bead, has in principle the same advantage as AFM in determining the contact area between the probe and the cell. When the trapped bead is pushed into the cell the contact can be estimated by the Hertzian contact model. In contrast to AFM, most experiments on cells using optical traps have been devised to measure their membrane tension, the bead is used to pull membrane tethers from the cell membrane [20]. The membrane tension depends on the bending rigidity of the lipid bilayer and its interaction with the actin cortex. Since we are interested in quantifying the cell’s mechanical response at small indentations, we focussed on the Young’s modulus. In conventionally configured optical traps the trapped bead is moved in the plane parallel to the coverslip (i.e. horizontally in the xy-plane) to pull or push on the sample. In this configuration the boundary conditions are not symmetric, and the interpretation of the results and the modelling of the experiment become more complicated.

To understand how the amount of deformation imposed on the cell affects its mechanical response, we established a method using an optical trap, which enabled us to manipulate the cell in the vertical direction (like an AFM) and to distinguish cellular responses upon forces below 10 pN. Using this technique, we were able to minimally indent the cells in the vertical direction, which enables us to calculate the Young’s modulus at small indentations (≈0.2 µm). To compare the mechanical response at larger (≈1 µm) indentations, we indented the cells with a bead that was attached to an AFM cantilever while applying forces of a few hundred pN.

We found that at low indentation (<0.2 µm) the cell showed an almost ideal elastic response, whereas at larger indentation the measured cell stiffness was dependent on the loading rate, following a weak power law. These results indicate that to extract mechanical properties such as the Young’s modulus and the response to dynamic perturbations, it is important to take the complex composition of the cell into account. Different cell mechanical properties can be accessed by changing the depth of indentation. A vertically operating optical trap together with AFM measurements provide the tools to measure surface-adhered cells at small and large indentations and enables the separation of viscous and elastic components of the cell.

## Results

### Cells Respond Elastic at Small Deformations and Viscous at Larger Deformations

**Figure 1:**
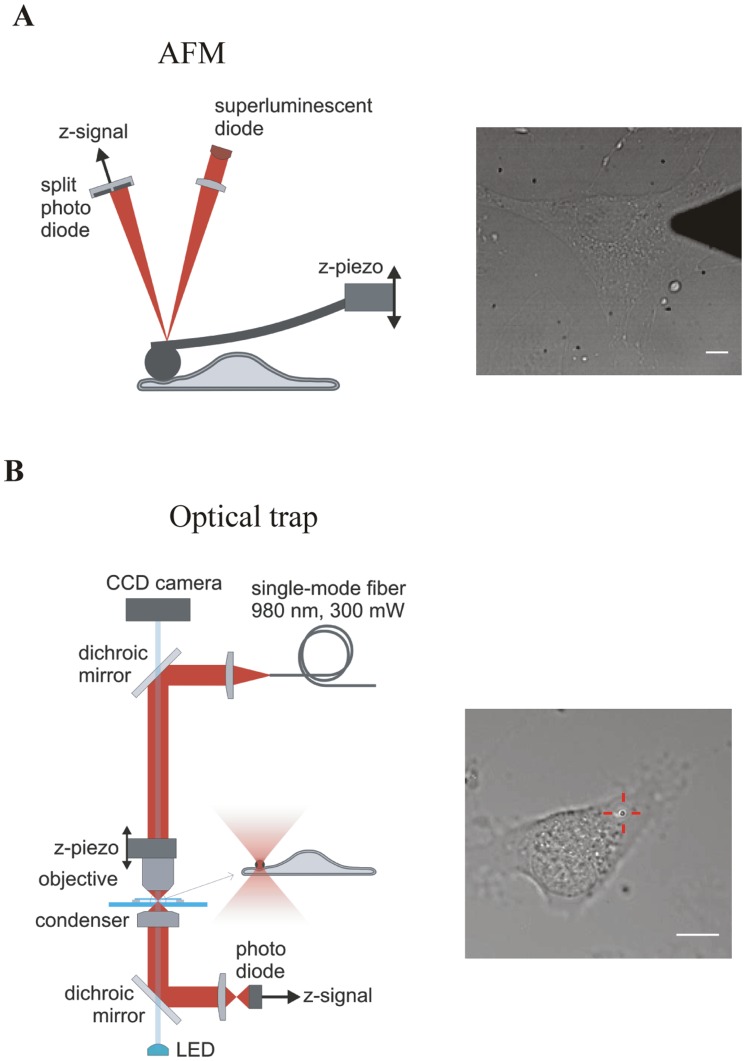
Experimental setups to measure the cell response in vertical direction. A) AFM: The cantilever is moved up and down with the z-piezo. When the AFM tip touches and indents the cell, the cantilever will bend. The amount of bending is measured via a laser beam that is reflected onto a split photodiode. Its electrical signal is linear proportional with the applied force on the cell. **B)** Optical trap: A laser beam, emitted from a single mode fibre, is coupled into the optical path of a standard upright microscope via a dichroic mirror and focused into the sample by the objective. The vertical position of the trap is controlled by a z-piezo that moves the objective up and down. The inset figure shows a bead that is trapped in the focus, and pushed into a cell. To monitor the displacement of the bead from the centre of the trap, the laser light is collected by the condenser, coupled out of the optical path via a second dichroic mirror and cast onto a photodiode. Both scale bars are 10 µm.

**Figure 2:**
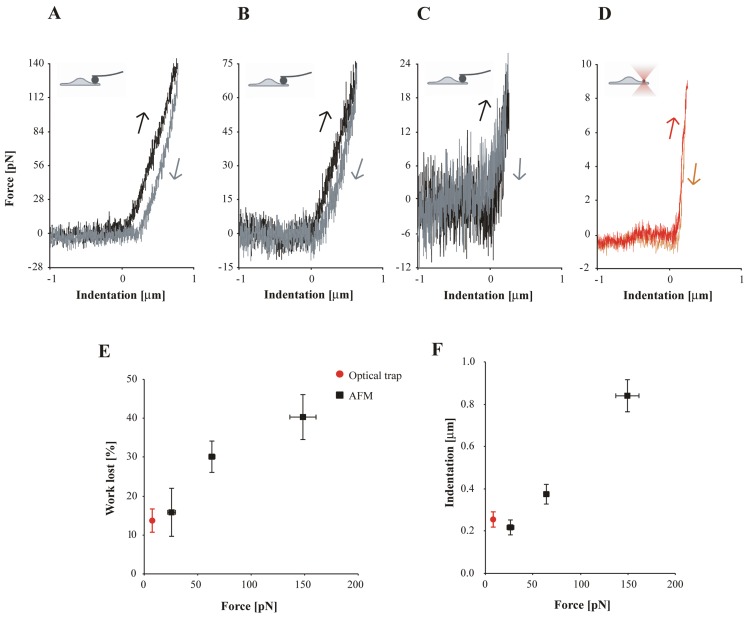
Sub-micron cell indentation with the optical trap and AFM. **A)** Cell indentation measured with AFM. The force was limited to 140 pN. The indentation (black) and retraction (grey) curves are not identical, but show a considerable amount of hysteresis. **B)** When the indentation force is limited to 75 pN the difference between the curves is reduced. **C)** At a force of 25 pN, close to the intrinsic noise of the AFM cantilever, hysteresis between the indentation and retraction curves cannot be clearly distinguished. **D)** Cell indentation measured with the optical trap. The high force resolution allows the controlled application of forces of less than 10 pN. The indentation (red) and retraction (orange) curves look identical with no obvious hysteresis. **E)** The relative amount of energy lost between indentation and retraction curves (mean ± s.e.m) was obtained by numerically calculating the area that is enclosed by the indentation and retraction curve, and dividing this by the area under the indentation curve. Only those measurements were analyzed that showed no sticking of the bead to the cell (which is clearly visible as a negative force during retraction). Each point represents measurements on 7 to 15 different cells. Both optical trap (red) and AFM (black) measurements that were performed at forces of up to 30 pN show that less than 15% of the indentation energy is lost. At higher forces this increased to almost 50%. **F)** The cell indentation was estimated for all indentation curves that were used for E). Data is shown as mean ± s.e.m. Measurements performed at the lowest forces (<30 pN) resulted at indentations of 0.2 µm, which increases to 0.8 µm at 150 pN. The contact point was defined as the position in the indentation curve where the force reaches a value below 0 pN (starting from the maximum force). The error in this method depends on the noise during the force measurement and will lead to an underestimation of the real indentation. Since the force noise in the AFM measurements is larger as compared to optical trapping also the error in the estimated indentation is larger.

We first investigated the cellular response at different forces by performing indentation measurements with a 1.98 µm diameter bead that was attached to an AFM cantilever (Fig. 1A). Under these conditions where a low force is distributed over a large area, we expect the induced cell damage to be negligible and the effects of the underlying substrate on the measured cell response to be small. If the response is elastic, the indentation and retraction curves will be identical. All the energy applied during indentation will be recovered during the retraction and none is lost. Figure 2A shows that there is a considerable difference between the indentation and retraction curve when a cell is indented with a force of 140 pN. This hysteresis shows that the response is not elastic. Most probably, viscous components are responsible for the dissipation of energy during indentation. We repeated the indentation experiments at lower forces. At a force of 75 pN the difference between the curves is less (Fig. 2B). Figure 2C shows that the applied force of 25 pN is almost on the same level as the noise, which makes these curves difficult to interpret. Lower forces could not be tested due to the thermal noise limitations in liquid.

To quantify the relative amount of energy that was lost due to hysteresis during the AFM experiments, we compared the energy that was required to indent the cells and the energy that was recovered during the retraction. Any loss of energy will be visible as a difference between the curves. To quantify for each experiment the difference between the indentation and retraction energy, we numerically calculated the integrals for all indentation and retraction curves. Figure 2E shows that the relative amount of work lost increased proportional with the force applied (from 15% at 30 pN to 40% at 150 pN).

To be able to measure the cellular response at lower forces we developed an optical trap that operates vertical with respect to the coverslip. Single cells were indented in the vertical direction with a 0.76 µm diameter bead that was held by the optical trap. The force is obtained by measuring the vertical displacement of the bead out of the centre of the focused laser beam (see methods and Fig. 1B). When the cell was indented at these low forces, the indentation and retraction curves showed little difference, indicating that the response was largely elastic (Fig. 2D). Figure 2E shows that in fact the amount of work lost during the indentation at 10 pN using the optical trap is 13%, which is less than the work lost during AFM experiments at the lowest possible indentation forces of ≈30 pN. A force of 30 pN applied with a 1.98 µm diameter bead corresponds to an already substantial cell indentation of approximately 0.2 µm (Fig. 2F). To determine the exact cell indentation, the contact point, the piezo position at which the bead touches the cell, needs to be detected. In this respect, the optical trap has the clear advantage of showing a lower force noise, which allows more accurate force spectroscopic experiments at lower forces. Using the optical trap, we can clearly distinguish forces as low as 1 pN, which allows us to estimate the cell indentation from ≈1 to ≈10 pN. The average indentation we measured on 90 different cells was 255±36 nm (avg. ± s.e.m) at an average force of 9.1±0.12 pN. The depth of indentation of the cell will also depend on the diameter of the bead that is used for the experiments (see also Eq. 1). The AFM experiments were performed with larger beads (1.98 vs. 0.76 µm) that are expected to induce ≈27% lower indentations at identical forces.

**Figure 3:**
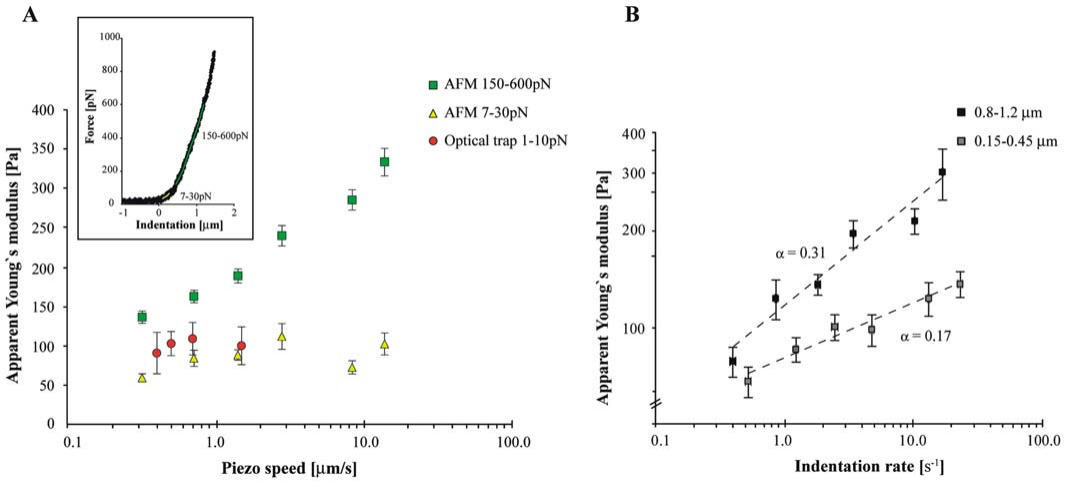
Cell stiffness at low and high indentation force at different indentation speeds. A) Young’s modulus of cells obtained with the optical trap (red) and AFM (yellow and green). For each speed 16 to 41 cells were measured. The data is plotted as function of the piezo speed (the actual cell deformation speed will be slightly lower and depends on the ratio between the cellular spring constant and cantilever spring constant). When the AFM indentation curves are fitted at 7–30 pN (yellow triangles), the obtained Young’s moduli are independent of the indentation speed. When the indentation curves are fitted at 150–600 pN (green squares) the apparent moduli increase a multifold at higher indentation speeds. **Inset)** AFM indentation curves were fitted between 7–30 pN (yellow) and 150–600 pN (green) using the Hertz model (Eq. 1). **B)** To compare the viscous contribution at the different indentation rates at an identical indentation of around 1 µm, we plotted the apparent modulus *k* as function of the indentation rate *f* of the experiment. This rate is defined as *f* = 1/(2*(*t*_1.2_-*t*_0.8_)), where *t*_1.2_-*t*_0.8_ is the time it took for the indentation to increase from 0.8 to 1.2 µm. The plot on a double logarithmic scale shows the fit to k(f) = A*f ^α^, where *α* = 0.31 (and *A* = 118). Similarly, we plotted the apparent modulus at an indentation of around 0.3 µm. The data still follows a power law, but with a reduced exponent of *α* = 0.17 (*A* = 80).

For elastic materials the measured response will be independent of the deformation rate. The experiments shown in Figure 2 were performed at a deformation rate of 0.8 µm/s. To determine, to what extent the cells respond elastically, we measured their response at varying deformation rates. We indented cells at a maximum force of 1 nN using the AFM at rates ranging from 0.3 to 14 µm/s. Since we found that at low forces the response showed very little hysteresis, we expect a predominantly elastic response of the cell at these low forces. We verified this by analyzing the cell response between 7–30 pN (Fig. 3A inset). We calculated the Young’s modulus *E* for the cell by fitting this 7–30 pN part using the Hertz model (Eq. 1, see methods). Figure 3A shows that for forces between 7 to 30 pN, the calculated Young’s modulus indeed hardly depends on the indentation rate. This confirms our earlier observation that for small deformations the cellular response is largely elastic. When we include the AFM measurements performed at different indentation rates and at forces up to 30 pN we obtained a modulus of 85.3±4.5 Pa (mean ± s.e.m., n = 237). In order to test whether similar results were found using the optical trap, we calculated the modulus (1–10 pN) and obtained a comparable elastic constant of 100.3±10.2 Pa (n = 90), showing that both techniques give consistent results for the Young’s modulus at forces at which the dissipated energy is negligible.

In contrast to elastic materials the response of viscous materials will strongly depend on the deformation rate, and the amount of hysteresis will increase at higher rates. When higher forces are applied, the cell will be indented deeper and viscous effects will increasingly contribute to the measured cell stiffness (Fig. 2E). In order to verify that at larger indentations the cell shows a viscous behaviour, we calculated the Young’s modulus using the obtained AFM data at higher forces (150–600 pN) and Eq. 1 (Fig. 3A inset). The obtained value for *E* increased from 140 Pa at low deformation rate to 330 Pa at high deformation rate. Our finding indicates that this *apparent* modulus is most likely a combination of the actual Young’s modulus and a viscous component that is responsible for the observed dependency on the deformation speed.

If at higher indentation the cell response is indeed viscous, then the increase in the apparent modulus can be used to estimate the viscous response of the part of the cell that is probed during indentation. However it has to be taken into consideration that if the apparent modulus increases at high deformation speed, the cell will be indented less deep at the analyzed force and the relative contribution of the viscosity will reduce (as shown in Fig. 2). To nevertheless obtain an estimate of the rheological properties of the cells we plotted the apparent modulus between an indentation of 0.8 and 1.2 µm as function of the indentation rate. Figure 3B shows that the increase in the apparent modulus follows a power law, with an exponent *α* = 0.31. From the results shown in Figure 2E and F we expect that this exponent should depend on the absolute indentation of the cell, and should decrease at smaller indentations. To verify this, we also analyzed the apparent Young’s modulus between an indentation of 0.15 and 0.45 µm. Figure 3B shows that the response still follows a power law, but now with a reduced slope of *α* = 0.17. This finding confirms that viscous effects have a stronger effect on the apparent modulus when the cell deformation gets larger.

### The Actin Cortex is the Major Contributor to the Measured Cell Elasticity at Small Deformations

The transition from elastic at small deformations to viscous at higher deformation shows that the elastic and viscous components are not homogeneously distributed in the cell. At low forces the probe will mainly deform the periphery whereas at higher forces the probe penetrates deeper into the cell and its more viscous interior will be measured. Predominant structures in the cell periphery are the lipid bilayer and the actin cortex. Using AFM measurements at high indentation forces, the cell response has been shown to depend on the actin cortex [24]. However, since this was tested at indentations in the micrometer range and actin networks themselves also show a viscous response [25], [26], it is interesting to investigate the role of the actin cortex on the cell elasticity at small deformations. We depleted cells of filamentous actin by treating them with Latrunculin-A (Lat-A). Lat-A binds to monomeric actin and thereby prevents polymerization into F-actin. Optical trap indentation experiments performed at forces of up to 10 pN showed that the Young’s modulus reduced from 100.3±10.2 Pa (n = 90) to 29.3±3.5 Pa (n = 45) after the addition of Lat-A. This effect shows that the F-actin network as part of the cell cortex is one of the major contributors to the cell elasticity at small deformations.

### The Cellular Elastic Response at Small Deformations is Anisotropic

**Figure 4:**
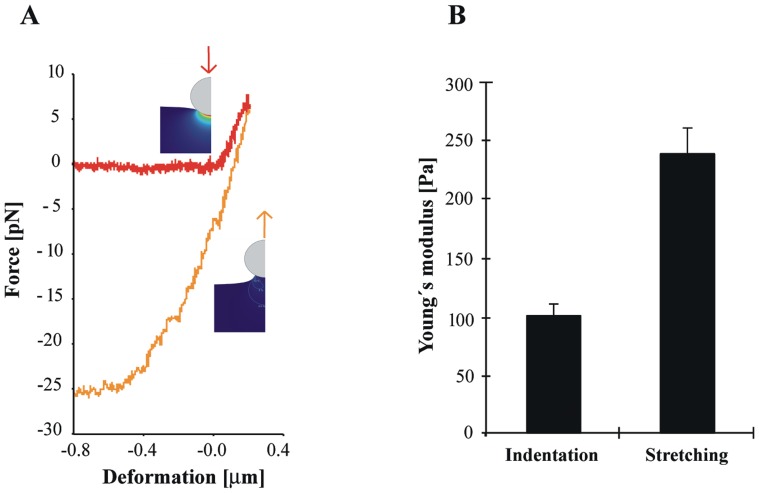
Cell stretching experiments. A) Beads that got stuck during cell indentation were used as a handle to stretch the cell. The stretching curve (orange) is steeper than the indentation curve. **B)** The Young’s modulus that is estimated from the stretching experiments is about twice as high (239 Pa) as the modulus we calculated from the indentation experiments (100 Pa).

For isotropic elastic materials the modulus is independent of the direction of deformation (indenting or stretching). In order to test to what extent the cell follows this ideal behaviour we performed stretching instead of indenting experiments on the cells at forces below 10 pN. We made use of the fact that in about 50% of all optical trapping experiments the bead got stuck to the cell and could therefore be used as a handle to stretch the cell (Fig. 4A). To derive a Young’s modulus from these experiments we used a variation on the Hertz model that describes the deformation of a large elastic body with a disc shaped contact area, which predicts a linear relation between force *F* and deformation *d_z_* (Eq. 3, see methods). Figure 4A shows that the retraction curve of a pulling experiment with a stuck bead is indeed close to linear. To obtain the relation between *F* and *d_z_* from the cell stretching experiments we performed a linear fit to the stretching part of the retraction curves (between -10 and 0 pN), and measured a slope of 88.5±8.27 µN/m (n = 21). In order to calculate the Young’s modulus we need to know the contact area. To obtain an estimate, we calculated the contact radius between the bead and the cell during the initial indentation. When the bead is pushed into the cell, *R_c_* will increase with the indentation (Eq. 2, see methods). Our indentation measurements showed an average indentation *d_z_* of 255 nm. Using *R_b_* = 380 nm this results in *R_c_* = 311 nm. Using Eq. 3, a Young’s modulus of 239±22 Pa was calculated for the cell stretching experiments (Fig. 4B), a value that is about twice as high as what we found from the indentation measurements. The actual modulus may be different as the contact radius may be larger or smaller. Larger, because we underestimate the indentation, as we only start to measure the indentation after it exceeds the thermal noise of ≈1pN, also adhesion effects between the bead and the cell are not included. Smaller, because the bead may not stick over the whole contact area. Still, even if we assume the error in the contact radius determination to be as high as 50%, the Young’s modulus will range from 160 to 480 Pa, always higher than what we extracted from the indentation experiments.

## Discussion

A variety of techniques have been employed during the last decades to measure cell mechanical responses upon stress. The obtained results have led to a picture of the cell as a complex composite material with both elastic and viscous components [27], [28]. Consequently the obtained cell response strongly depends on the experimental method and timescale.

To study the cell mechanical responses at very small deformations we constructed a vertically operating optical trap that allowed the application of forces of less than 10 pN, which will minimally disturb the physiological condition of the cell. The main difference as compared to the AFM is that the cantilever is replaced by a focused laser beam to hold the bead. With the optical trap a much lower spring constant is achieved (1^−4^ N/m compared to 0.08 N/m for the AFM). The clear advantage of the optical trapping experiments is that the force noise is much lower due to the absence of a large cantilever, which allows more accurate force spectroscopic experiments at lower forces. The noise levels of conventional AFM cantilevers are around 20 pN [10], which makes it difficult to perform and analyze AFM indentation experiments at low forces. Due to the noise in the force signal the point of contact in indentation experiments is difficult to determine during AFM experiments. In contrast, the optical trap described here can be used to distinguish forces as low as 1 pN. This allows measurements of the cellular response at forces between 1 to 10 pN, which is comparable to the force a few motor proteins can exert. Laser optical traps are widely used to measure mechanical properties of biological systems, and in most experiments the plane of exerting and measuring the forces is parallel to the coverslip. This limits such experiments to cells that extend far enough from the coverslip such that a bead can be attached to its side. Pulling in the direction vertical with respect to the coverslip allows this technique to be applicable to a wider range of cell types, including flat cells. The cells are effectively compressed or stretched between the surface on one side and the bead on the other. As a result the boundary conditions are symmetric, which greatly simplifies the modelling of the cell deformation. Alternatively, a symmetric condition can also be achieved by using dual optical traps to stretch suspended cells in the xy-plane [29], [30]. It should be noted, that although during AFM experiments the force is also applied in a vertical direction, the AFM cantilever is bending during the indentation experiment, resulting in an additional horizontal component of the applied force. Taken together, both the vertical operation of the optical trap as well as the low force noise regime enabled us to investigate the mechanical response of cells at very low forces.

Using the optical trap we obtained a Young’s modulus of 100 Pa for fibroblasts. Previous measurements of the Young’s modulus using AFM had reported values that were 10 to 100-times higher [24], [31]. We validated our optical trapping experiments by analyzing the 7–30 pN part of AFM indentation experiments and obtained an *E* of 85 Pa. These results show clearly that both experimental methods give comparable results for the obtained elasticity. The discrepancy between our and previous AFM indentation results can for a large part be explained by the increase in stiffness that occurs at higher indentations, which is due to viscous effects. Our measurements show that the elastic response of the cell is limited to small deformations up to ≈0.2 µm. Both, deforming the cells at varying deformation rates as well as the fact that we observed almost no hysteresis between the retraction and indentation measurements indicate that at this deformation regime, the cell behaves largely elastic. This ideal elastic behaviour seems to break down, at least in part, when we stretched the cell. Although the exact boundary conditions during the pulling experiments are more difficult to determine (contact area, adhesion), the estimated Young’s modulus is approximately twice as high as compared to what we found in the indentation experiments. A recent report, where lung fibroblast were indented and stretched with a flat-ended AFM tip, showed also an anisotropic response but in the opposite direction [32]. Upon stretching, these cells were 6 times softer as compared to indentation. An obvious difference with our experiments is that the induced deformations were larger (≈1 µm), which could have led to a larger role of the viscosity in these experiments.

Previous work by Rotsch and Radmacher showed that the cell’s mechanical response is determined to a large extend by the underlying cytoskeleton, when indented by AFM in the range of a few micrometers [24]. Depolymerization of actin filaments and (actin based) stress fibers resulted in a reduction of the cell stiffness, whereas the microtubule network hardly affected the measured cell stiffness. Our indentation experiments at low forces in which we mainly probed the elastic components of the cell show that also the Young’s modulus is largely determined by the actin cortex.

As soon the cell is deformed for more than a few hundred nanometres the cell response is increasingly determined by viscous effects. Larger deformations will be much more sensitive to the cell interior, the cytoplasm and cell nucleus and the remodelling of the actin cytoskeleton will start to play a role. This results in an increase of the apparent Young’s modulus and a hysteresis between the indentation and retraction curves, both of which depend on the rate of deformation. This is also evident from multiple rheology experiments on cells that have showed that the frequency dependent stiffness of the cells follows a weak power law. A recent review on this topic showed that the majority of these experiments that probed the outermost layer of the cells at small deformations yielded values for the exponent *α = *0.13–0.17, whereas measurements that deformed the whole cell at larger deformations gave *α = *0.24–0.29 [27]. This finding implies that by studying the qualitative frequency response, the cell’s cortex can be distinguished from its cytosolic interior.

Also in our measurements the apparent modulus followed a weak power law, which shows that the cell’s viscosity is not constant (else *α* = 1) but decreases at higher deformation rates. Within a single AFM indentation experiment one can simply differentiate between small and large deformations by analyzing different parts of the indentation curves. Thus, within a single set of measurements we found *α = *0.17 for deformations of ≈0.3 µm, and *α = *0.31 for larger deformations of ≈1 µm. Both values are consistent with values described in the literature for small and large deformations [27]. From our optical trapping data we found no significant effect of the indentation speed on the measured cell stiffness at low forces. It would be interesting to be able to extend the frequency range of the optical trapping experiments to quantify *α* at forces below 10 pN.

Our results demonstrate that both AFM and vertical optical laser trapping provide consistent values for the cell’s Young’s modulus, provided that the cell deformation is small. As optical trapping experiments are generally carried out at much lower forces than AFM experiments, care has to be taken when interpreting measurements obtained with different techniques. When they are not analyzed at the same magnitude of force, optical trapping experiments will give lower values for the cell’s elastic modulus than measurements on cells using AFM [33].

In conclusion we showed that a vertically operated optical trap provides an alternative method to characterize the elastic response of cells at small deformations. Due to the reduced force noise the contact point and the absolute indentation of the cell can be more accurately determined than in AFM measurements. Perturbation of the physiological condition of the tested cell is avoided by the application of forces that can be limited to single pNs. The induced indentations are small, which minimizes the influence of the underlying substrate. This low force approach will be helpful in determining the elastic response of a wide range of cells including fragile and relatively flat cells. In combination with AFM measurements this will be useful to understand the properties of the cellular composite material in more detail.

## Materials and Methods

### Sample Preparation

3T3 mouse embryonic fibroblasts (DSMZ, Braunschweig, Germany) were grown in Dulbecco’s modified Eagle’s medium (DMEM) supplemented with 10% fetal bovine serum, and Penicillin/streptomycin (Lonza Cologne AG, Cologne, Germany). At least 24 hours before the experiment, cells were removed from the culture flasks with 0.25% trypsin/EDTA (Invitrogen, Darmstadt, Germany) and seeded onto poly-L lysine coated coverslips. Before the experiment, a coverslip containing the cells was briefly washed with modified Krebs’-Ringer solution (120 mM NaCl, 4.7 mM KCl, 1.2 mM CaCl_2_, 0.7 mM MgSO_4_, 10 mM Glucose, 10 mM Na-Hepes, pH 7.4) [34]. The cells were imaged in the same buffer. For disruption of the actin cortex, the cells were incubated with 1 µM Latrunculin-A (Lat-A, Calbiochem, Darmstadt, Germany) for 30 min and measured directly afterward. All experiments were performed at room temperature. The part of the cell that was chosen for indentation was between the nucleus and the periphery. Both AFM and optical trapping measurements were performed at approximately similar positions on the cells.

### AFM

AFM indentation experiments were carried out with a MFP-3D (Asylum Research, Santa Barbara, CA, USA), that was mounted on a custom built inverted optical microscope using an oil immersion objective (60×1.45NA plapon objective, Olympus, Japan). A coverslip containing the cells was mounted in an open sample chamber. First a cell was selected using the optical microscope. The AFM tip was brought down to indent the cell, during which the motion of the z-piezo and the applied force were recorded (Fig. 1A). The cell deformation was computed from the displacement of the z-piezo minus the bending of the cantilever. In preliminary experiments with conventional AFM tips (pyramidal shape with a 20 nm radius at its apex), the cells often showed blebbing minutes after the cell was probed.

This problem was successfully solved by using cantilevers with a 1.98 µm diameter bead glued to the end (CP-PNPL-PS, NanoAndMore GmbH, Wetzlar, Germany). Under these conditions where a low force is distributed over a large area, we expect the induced cell damage to be negligible and the effects of the underlying substrate on the measured cell response to be small. To be able to detect and to apply low forces, we used cantilevers with a low spring constant of 0.08 N/m. All cantilevers were individually calibrated by fitting the power spectrum to a simple harmonic oscillator [35]. The calibrated force curves were window averaged to 1 kHz.

### Optical Trap

The optical trap that was used to indent the cells at low force was built around a commercial upright microscope body (Eclipse 50i, Nikon, Japan) and set up for trapping and detection vertical to the coverslip (Fig. 1B). The 980 nm laser light emitted from a 300 mW single mode fibre was collimated and combined with the optical path using a dichroic mirror (unless specified all optical components were purchased at Thorlabs GmbH, Germany). The laser beam was focused in the sample by a water immersion objective (60×1.27NA Plan Apo IR objective, Nikon, Japan). A closed-loop objective-piezo element (P-721, Physik Instrumente GmbH, Karlsruhe, Germany) was used to move the objective up and down. To measure the position of the trapped bead with respect to the trap centre, the trapping laser light was collected through an air condenser and projected onto a quadrant photo detector placed in a conjugate plane of the back focal plane. The detector was overfilled to achieve an effective numerical aperture of 0.4 for the collection of the laser light [36]. The sum signal of the quadrant photo detector that contains the z-position information of the trapped bead was digitized by an analogue to digital converter at a sampling frequency of 12 kHz (NI USB-6212, National Instruments, Austin, TX, US). The imaging part of the microscope consisted of a blue LED that was imaged on the back focal plane of the condenser and a CCD camera placed at 200 mm from the tube lens.

A coverslip containing the cells was mounted in a closed sample chamber, consisting of a microscope slide, a 100 µm thick spacer and the coverslip. Before the sample chamber was closed, polystyrene beads of 0.76 µm diameter (Bangs laboratories, Fishers, IN, USA) were added to the buffer.

Although the trapping laser is focused onto the bead and outside of the cell, most of the laser light will pass through the cell for the duration of the experiment (Fig. 1B). To minimize the potential cell damage we used near-infrared light. The used wavelength of 980 nm was specifically chosen to minimize the induced cell damage [37]. To confirm this we positioned the laser focus just outside of the cell periphery for 10 minutes (about 10 times the duration of the indentation experiment) and monitored the cell for signs of stress using optical microscopy. One hour after the exposure to the laser light, all cells (n = 6) were completely indistinguishable from the non-radiated cells, with no signs of bleb formation or other changes in cell appearance.

### Calibration of the Optical Trap

The trap stiffness and the detector response were calibrated by recording the power spectrum of the position signal of the trapped bead and applying the equipartition theorem [38]. We trapped a bead and recorded its noise during its approach towards the cell and performed the calibration at intervals of 1 µm. By using a water immersion an almost constant trap stiffness can be achieved [39]. For the cell indentation experiments we used the calibration value that was recorded at a height of 2 µm above the cell. During the approach a feedback loop was used to detect the contact of the bead with the cell. Once a force higher than ≈10 pN was detected the approach was stopped automatically. To test the linear range of the trap in z-direction we focused the trapping laser on a bead that was bound to the coverslip and moved the trap in the z-direction with a 3 µm triangular wave. For displacements of up to 500 nm out of the trap centre, the signal was linearly proportional to the displacement (not shown). The axial spring constant of the trap was >0.1 pN/nm, which gave a maximum force that could be reliably measured on the cell of 50 pN, which was sufficient for our measurements. The calibrated traces were window averaged to 1 kHz and showed the displacement of the bead from the centre of the trap. The force was calculated by multiplying the displacement with the trap stiffness. The cell deformation was computed from the displacement of the trap focus minus the displacement of the bead.

### Calculation of the Young’s Modulus

To calculate the Young’s modulus *E* from our indentation experiments we used the Hertz model that describes the indentation of a large elastic body with a rigid spherical indenter:
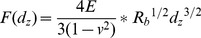
(1)


Eq. 1 predicts the force *F* to increase exponentially with the indentation *d_z_*. *R_b_* is the bead radius and *v* the Poisson’s ratio for which we choose 0.4 [40]. When the bead is pushed into the cell, the contact radius *R_c_* will increase with the indentation:
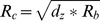
(2)


To calculate the Young’s modulus from our stretching experiments we used a variation on Eq. 1 that describes the deformation of a large elastic body with a disc shaped contact area [41]:
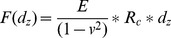
(3)


*R_c_* is the radius of the disc shaped contact area between the bead and the cell. As long as this radius is constant the relation between *F* and *d_z_* will be linear.
